# Food insecurity, habits, and working conditions in subsistence workers during COVID-19 isolation, Medellin, Colombia, 2021

**DOI:** 10.47626/1679-4435-2024-1279

**Published:** 2026-07-06

**Authors:** María Osley Garzón-Duque, Fabio León Rodriguez-Ospina, Sara Toro Tobón, Elsa María Vasquez-Trespalacios, Valentina Martinez Naranjo

**Affiliations:** 1 Facultad de Medicina, Universidad CES, Medellín, Antioquia, Colombia; 2 Facultad Nacional de Salud Pública, Universidad de Antioquia, Medellín, Antioquia, Colombia

**Keywords:** working conditions, food insecurity, COVID-19, eating habits, informal sector.

## Abstract

**Introduction:**

Mandatory isolation and quarantine measures implemented between 2020 and 2021 had important repercussions on labor dynamics.

**Objectives:**

To identify the characteristics of COVID-19 isolation, as well as the habits and working conditions that characterize food insecurity in subsistence workers in Medellin, Colombia, in 2021.

**Methods:**

Cross-sectional study using primary data sources. An interviewer-administered survey was conducted among 656 workers selected through snowball sampling.

**Results:**

Overall, 56.4% were men and 74.7% were aged ≥45 years. Furthermore, 89.9% of women were the primary household income provider, and 74.8% did not have a partner. Alcohol and tobacco use were more frequent among men, and 43.0% of women consumed two meals a day. Furthermore, 95.6% of participants underwent mandatory quarantine; 73.2% remained isolated > 12 weeks; and 77.0% received support during isolation. The prevalence of moderate/severe food insecurity was 44.1%, increasing to 51.4% among women. Conditions characterizing food insecurity included being female, consuming one meal a day, not having a partner, being the primary household income provider, lacking work permit, not receiving government food assistance, making payment arrangements with landlords, and receiving food assistance from a university.

**Conclusions:**

Although food insecurity decreased by 10 percentage points, the conditions characterizing it reveal the subsistence circumstances experienced by workers, particularly women, whose social and occupational disadvantage deepened.

## INTRODUCTION

Informal work in Colombia is a growing phenomenon. According to the Organisation for Economic Co-operation and Development (OECD), it accounted for 49.9% in 2019 and reached 53.1% of total employment in 2021 [^1^]. This employment modality is prevalent among populations with limited educational attainment, weak integration into the formal economy, and restricted access to the general social security system [^2^]. These individuals often engage in subsistence activities [^3^], as is the case of perishable and non-perishable goods on urban streets and sidewalks, who in Medellín, Colombia, are referred to as *“venteros”*. During the pandemic, this group experienced a marked deterioration in their living conditions, with increased socioeconomic, family, food, and nutritional vulnerability as a result of mandatory lockdown measures and the suspension or reduction of their sales, even after returning to the streets to resume their activities.

This situation may have influenced eating habits, lifestyle, and household food insecurity. However, these conditions have not been clearly explored or sufficiently documented. Although the profile of food and nutritional insecurity in this working group has been identified in the pre-pandemic period [^4^], with a prevalence of 53.9%, factors associated with moderate to severe food insecurity (MSFI) were observed, such as abstaining from alcohol consumption, mood-related appetite disturbances, consuming one or two meals a day without a defined eating schedule, lack of work permit, and female sex. These conditions were, in turn, associated with sedentary behavior, high intake of fatand carbohydrate-rich foods, and the presence of overweight, obesity, diabetes, and hypertension [^5^-^7^].

Despite the foregoing, there is still limited evidence describing the profile of food and nutritional insecurity experienced during the pandemic. This profile may be partly determined by their sociodemographic and working conditions, as well as by isolation circumstances and dietary habits adopted both during and after mandatory lockdown. In the context of the COVID-19 pandemic, the socioeconomic and food-related vulnerabilities of individuals relying on subsistence employment may have been exacerbated [^8^,^9^].

Mandatory isolation and quarantine measures implemented between 2020 and 2021 had significant repercussions on labor dynamics, initially causing the interruption of work activities [^10^] and subsequently affecting food sovereignty on a global scale [^11^,^12^]. This was notably reflected in dietary habits and levels of food insecurity, driven by reduced food availability in the market, increased food prices, and a noticeable reduction in household income [^12^-^15^].

In Latin America and the Caribbean, data from the Panamerican Health Organization (PAHO) indicate a sustained increase in levels of hunger and food insecurity since 2015, with a more pronounced rise beginning in 2019. By 2020, the prevalence of hunger reached 9.1%, representing an increase of two percentage points. Additionally, a 9.0 percentage point increase in moderate to severe insecurity was observed in the region [^16^].

For the reasons outlined above, this study identified the characteristics of COVID-19 related isolation, dietary habits, and working conditions that shape MSFI among subsistence workers in Medellín, Colombia in 2021. The aim was to provide evidence to support the planning and implementation of public health and occupational health interventions, including food and nutritional security measures for these populations in the context of emergencies, natural or human-made disasters, and pandemics.

## METHODS

### POPULATION AND SAMPLE

**Figure 1:**
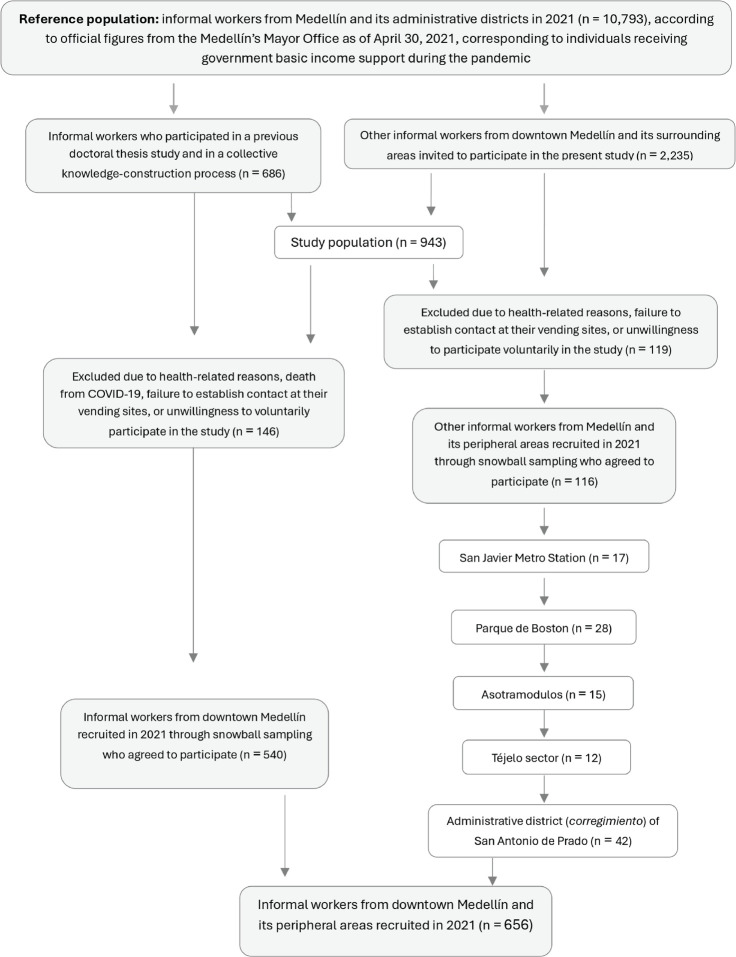
Study population selection process. Source: Prepared by the authors using their own data.

A total of 656 informal workers from the city of Medellín and the administrative district (*corregimiento*) of San Antonio de Prado were recruited using snowball sampling (Figure 1). Participants were invited by their leaders and the principal investigator at their vending sites, as well as during meetings and trade association assemblies. A structured, interviewer-administered survey was conducted following prior face and content validation with a group of leaders and workers.

### INCLUSION CRITERIA

Individuals aged over 18 years, with at least 3 years of occupational experience, who were informed about the study and its procedures, agreed to participate, and provided written consent prior to data collection.

### VARIABLES

Dependent variable: MSFI, used to screen households at risk of food insecurity, according to the Latin American and the Caribbean Food Security Scale [^17^], a 15-item instrument with dichotomous (yes/no) responses. The following cut-off points were considered: zero for food security; one to five for mild insecurity; six to ten for moderate insecurity; and eleven to fifteen for severe insecurity. For bivariate and multivariate analysis, categories were grouped as moderate/severe food insecurity (6 to 15 points) and food security/mild insecurity (zero to five points).

Explanatory variables included sex; age (recategorized into two and four groups); primary household income earner; marital status; and work permit. Variables related to lifestyle and dietary habits included physical activity level; alcohol and tobacco use; meal timing; food origin; eating companionship (alone or with others); relationship between emotional state and food consumption; time food remained at the workplace before consumption; number of meals a day; concurrent activities during food intake; and cooking methods. Mandatory isolation and support variables included compliance with isolation; duration of isolation in weeks; receipt of support; receipt of government food assistance; type of support received; provision of support during isolation, duration of support; receipt of government subsidies during the pandemic; and need for government support.

### CONTROL OF ERRORS AND BIASES

Multivariate statistical analyses were adjusted for sex, age, and occupational tenure. Selection bias was controlled by including workers who agreed to participate and met the inclusion criteria. Information bias was controlled through the use of an instrument validated for face and content, awareness activities with workers and leaders, standardization of investigators and the interviewer, and refinement of both the instrument and data collection after a pilot test involving eight workers.

### STATISTICAL ANALYSIS

Descriptive analyses were conducted using absolute frequencies and percentages for all variables. Bivariate and multivariate analyses were also performed to explore associations between independent variables and MSFI. Bivariate analysis was conducted using chi-squared tests and prevalence ratios (PRs) with 95% confidence intervals (95%CIs). Multivariate analysis was performed using binomial regression to identify conditions associated with MSFI in workers’ households. Variables with p-values < 0.25 were entered sequentially into the model in ascending order of p-value.

Interdependent multivariate analysis was performed using multiple correspondence analysis to characterize the profile of food insecurity, incorporating variables with explanatory capacity identified in dependent multivariate analyses, whose associations were verified using chi-squared test. The following variables were included: primary household income earner, age, marital status, number of meals a day, work permit, duration of support, government food assistance, payment arrangement with the landlord of the dwelling, and sex. MSFI was included as a supplementary variable. Statistical analyses were performed with a 95.0% confidence level and 5.0% significance level, using SPSS^®^ version 26 (Universidad de Antioquia license) and Epidat version 3.1. Tables and text formatting were prepared using Microsoft Word.

### ETHICAL CONSIDERATIONS

This article presents the results of the nutritional and food-related component of the main project: *“*Living, working, and health conditions among a group of informal workers (“*venteros*”) in Medellín during the pandemic and post-pandemic period, 2021-2022,” approved by the Institutional Research Ethics Committee of Universidad CES, under Minutes No.156 dated February 3, 2021. The study was classified as posing minimal risk in accordance with Resolution 8,430, and international guidelines for research involving vulnerable populations developed by the Council for International Organizations of Medical Sciences.

## RESULTS

### SOCIODEMOGRAPHIC CONDITIONS

Men accounted for the majority of participants (56.4%), and 74.7% were aged ≥ 45 years. A higher concentration of women was observed among individuals aged 45-59 years, whereas men predominated among those aged ≥ 60 years. More than 85.0% were the primary household income earner, with a higher prevalence among women. Furthermore, 56.4% did not have a partner, a condition more frequent among women (74.8%). In addition, 50.8% lacked work permit, reaching 54.1% among men (Table 1).

**Table 1:** Descriptive statistics of sociodemographic conditions, dietary habits, lifestyles, isolation during quarantine, support received, and food insecurity among workers participating in the study (n = 656)

Condition/characteristic	Sex				Total	
	Male		Female			
	n	%	n	%	n	%
Sociodemographic conditions						
Age (four age groups)						
18-29 years	10	2.7	14	4.9	24	3.7
30-44 years	62	16.8	80	28.0	142	21.6
45-59 years	155	41.9	127	44.4	282	43.0
≥ 60 years	143	38.6	65	22.7	208	31.7
Age (two age groups)						
18-49 years	113	32.3	132	46.1	245	38.5
≥ 50 years	237	67.7	154	53.8	391	61.5
Primary household income earner (n = 655)						
Yes	301	81.6	257	89.9	558	85.2
Marital status						
Without a partner	156	42.2	214	74.8	370	56.4
With a partner	214	57.8	72	25.2	286	43.6
Work permit						
Yes	200	54.1	133	46.5	333	50.8
Lifestyles and dietary habits						
Physical activity level						
Sedentary	54	14.6	64	22.4	118	18.0
Little active	41	11.1	34	11.9	75	11.4
Active	179	48.4	106	37.1	285	43.4
Very active	96	25.9	82	28.7	178	27.1
Alcohol use (n = 655)						
Yes	102	27.6	35	12.2	137	20.9
Tobacco use (n = 655)						
Yes	82	22.2	43	15.1	125	19.1
Defined eating schedule						
Yes	174	47.0	90	31.5	264	40.2
Consumption of vitamins						
Yes	101	27.30	91	31.82	192	29.27
Origin of the food consumed						
Household	263	71.1	252	88.1	515	78.5
Restaurant	189	51.1	128	44.7	317	48.3
Eating companionship						
Alone	166	44.9	107	37.4	273	41.6
With others	204	55.1	179	62.6	383	58.4
Mood state affects food consumption (n = 655)						
Yes	93	25.2	130	45.4	223	34.0
Time food remains at the vending site before consumption						
< 1 hour	183	49.7	106	37.2	289	44.3
1-3 hours	157	42.7	157	55.1	314	48.1
4-5 hours	25	6.8	19	6.7	44	6.7
> 5 hours	3	0.8	3	1.0	6	0.9
Number of meals a day						
1	11	3.0	20	7.0	31	4.7
2	108	29.2	123	43.0	231	35.2
3	224	60.5	119	41.6	343	52.3
> 3	27	7.3	24	8.4	51	7.8
Concurrent activities during food intake						
Devotes exclusive time for eating	120	32.43	66	23.08	186	28.35
Combines meals with serving customers	249	67.3	221	77.3	470	71.7
Combines meals with handling money	164	44.3	102	35.7	266	40.6
Cooking methods						
Baked	137	37.3	114	39.9	251	38.3
Grilled	132	35.7	116	40.6	248	37.8
Fried	272	73.5	181	63.3	453	69.1
Steamed	121	32.7	108	37.8	229	34.9
Isolation and support received						
Compliance with mandatory isolation						
Yes	347	93.8	281	97.9	628	95.6
Duration of isolation in weeks						
≤ 4	33	9.5	24	8.5	57	9.1
5-8	29	8.4	11	3.9	40	6.4
9-12	43	12.4	28	10.0	71	11.3
> 12	242	69.7	218	77.6	460	73.2
Received support during mandatory isolation (n = 652)						
Yes	274	74.7	228	80.0	502	77.0
Government subsidies during quarantine/pandemic (n = 654)						
Yes	211	57.0	182	64.1	393	60.1
Need for government support						
Yes	330	89.2	262	91.6	592	90.2
Type of support received during mandatory isolation (n = 506)						
Financial	105	38.2	84	36.4	189	37.3
Food assistance	142	51.6	137	59.31	279	55.1
Housing	3	1.1	1	0.4	4	0.8
Payment arrangement with the landlord of the dwelling	39	14.2	37	16.0	76	15.0
Source of support during mandatory isolation						
National agency	31	15.1	12	6.6	43	11.1
Local university	164	80.0	151	83.4	315	81.6
Workers’ association	3	1.5	1	0.6	4	1.0
Other	7	3.4	17	9.4	24	6.2
Duration of support in months						
≤ 1	97	35.3	56	24.6	153	30.3
2-3	156	56.7	156	67.8	312	61.8
4-5	17	6.2	12	5.2	29	5.7
≥ 6	5	1.8	6	2.6	11	2.2
Household food insecurity						
Moderate/severe	142	38.4	147	51.4	289	44.1
Absent/mild	228	61.6	139	48.6	367	55.9

### SOCIODEMOGRAPHIC AND LIFESTYLE CONDITIONS ASSOCIATED WITH FOOD INSECURITY

Statistically significant associations (p < 0.05) were identified between MSFI and biological sex, age, marital status, work permit, and tobacco use. The prevalence of MSFI was 34.0% higher among women (PR = 1.34; 95%CI = 1.13-1.60), 28.0% higher among individuals aged 18-49 years (PR = 1.28; 95%CI = 1.05-1.56), 16.0% higher among those without a partner (PR = 1.16; 95%CI = 1.01-1.33), 26.0% higher among those lacking work permit (PR = 1.26; 95%CI = 1.08-1.47), and 37.0% higher among smokers (PR = 1.37; 95%CI = 1.00;1.88) (Table 2).

**Table 2:** Sociodemographic conditions, lifestyles, dietary habits, isolation, and support received during the pandemic associated with moderate/severe food insecurity in the households of workers participating in the study (n = 656), Medellín, 2021

Condition/characteristic	Food insecurity				Total	Chi-squared (p-value)	PR (95%CI)
	Moderate/severe		Absent/mild				
	(n%)		(n%)				
Sociodemographic conditions							
Sex							
Female	147	50.87	139	37.87	286	11.09 (0.000)	1.34 (1.13-1.60)
Male	142	49.13	228	62.13	370		1.00
Age (two age groups)							
18-49 years	123	42.56	122	33.24	245	5.61 (0.017)	1.28 (1.05-1.56)
≥ 50 years	166	57.44	245	66.76	411		1.00
Main household income earner							
Yes	253	87.85	305	83.11	558	2.87 (0.090)	1.05 (0.99-1.12)
No	35	12.15	62	16.89	97		1.00
Marital status							
Without a partner	177	61.25	193	52.59	370	4.92 (0.026)	1.16 (1.01-1.33)
With a partner	112	38.75	174	47.41	286		1.00
Work permit							
No	161	55.71	162	44.14	323	8.65 (0.003)	1.26 (1.08-1.47)
Yes	128	44.29	205	55.86	333		1.00
Lifestyles							
Physical activity							
Sedentary	53	18.34	65	17.71	118	4.33 (0.228)	0.96 (0.75-1.24)
Little active	39	13.49	36	9.81	75		1.12 (0.85-1.46)
Active	114	39.45	171	46.59	285		0.86 (0.69-1.06)
Very active	83	28.72	95	25.89	178		1.00
Alcohol use							
Yes	54	18.75	83	22.62	137	1.46 (0.227)	0.83 (0.61-1.12)
No	234	81.25	284	77.38	518		1.00
Tobacco use							
Yes	65	22.49	60	16.39	125	3.89 (0.048)	1.37 (1.00-1.88)
No	224	77.51	306	83.61	530		1.00
Dietary habits							
Defined eating schedule							
Yes	103	35.64	161	43.87	264	4.55 (0.032)	0.81 (0.67-0.99)
No	186	64.36	206	56.13	392		1.00
Consumes meals at the workplace							
Yes	212	73.61	314	85.79	526	15.19 (0.000)	0.68 (0.57-0.81)
No	76	26.39	52	14.21	128		1.00
Mood state affects food intake							
Yes	127	43.94	96	26.23	223	22.57 (0.000)	1.67 (1.35-2.07)
No	162	56.06	270	73.77	432		1.00
Time food remained at the workplace before consumption							
≥ 3 hours	27	14.36	23	6.30	50	9.80 (0.002)	2.28 (1.34-3.86)
< 3 hours	161	85.64	342	93.70	503		1.00
Number of meals a day							
1	23	7.96	8	2.18	31	50.78 (0.000)	3.44 (1.96-6.04)
2	134	46.37	97	26.43	231		2.69 (1.57-4.59)
3	121	41.87	222	60.49	343		1.63 (0.95-2.81)
> 3	11	3.81	40	10.90	51		1.00
Devotes exclusive time for eating							
Yes	72	24.91	114	31.06	186	3.01 (0.083)	0.84 (0.68-1.03)
No	217	75.09	253	68.94	470		1.00
Combines meals with serving customers							
Yes	215	74.39	255	69.48	470	1.92 (0.166)	1.15 (0.94-1.41)
No	74	25.61	112	30.52	186		1.00
Conditions of COVID-19 isolation and support received during the pandemic							
Underwent mandatory quarantine							
Yes	275	94.83	352	95.91	627	0.44 (0.508)	0.87 (0.61-1.27)
No	15	5.17	15	4.09	30		1.00
Duration of isolation in weeks							
≤ 4	26	9.45	30	8.52	56	0.492 (0.920)	1.08 (0.82-1.41)
5-8	18	6.55	22	6.25	40		1.04 (0.73-1.50)
9-12	33	12.00	38	10.80	71		1.08 (0.82-1.41)
> 12	198	72.00	262	74.43	460		1.00
Duration of support during the pandemic in months							
≤ 1	78	34.98	75	26.60	153	4.24 (0.236)	1.40 (0.63-3.11)
2-3	129	57.85	183	64.89	312		1.14 (0.51-2.51)
4-5	12	5.38	17	6.03	29		1.14 (0.46-2.78)
≥ 6	4	1.79	7	2.48	11		1.00
Type of support received							
Financial							
Yes	77	34.53	112	39.58	189	1.36 (0.244)	0.87 (0.69-1.10)
No	146	65.47	171	60.42	317		1.00
Food assistance							
Yes	124	55.61	155	54.77	279	0.03 (0.851)	1.02 (0.87-1.19)
No	99	44.39	128	45.23	227		1.00
Housing							
Yes	2	0.90	2	0.71	4	0.06 (0.810)	1.27 (0.18-8.94)
No	221	99.10	281	99.29	502		1.00
Payment arrangement with the landlord of the dwelling							
Yes	49	21.97	27	9.57	76	14.97 (0.000)	2.29 (1.48-3.54)
No	174	78.03	255	90.43	429		1.0
Government subsidies							
Yes	162	56.25	231	63.11	393	3.17 (0.075)	0.89 (0.78-1.01)
No	126	43.75	135	36.89	261		1.00

* Statistically significant association when p < 0.05.

Bold type denotes statistical significance.

### LIFESTYLE AND DIETARY HABITS

More than 70.0% of the sample was physically active or very active, predominantly men, whereas 22.4% of women were sedentary. Alcohol and tobacco use were more frequent among men (27.6% and 22.2%, respectively). Moreover, 78.5% consumed food brought from home, a practice more common among women (88.1%), and 45 out of every 100 men consumed their meals alone. In addition, 34.0% reported that their mood state affected food intake, with women being more affected (45.4%). Most participants kept food at the vending site for ≤ 3 hours before consumption, and for half of the men this duration was < 1 hour (49.7%) (Table 1).

Although 87.5% consumed between two and three meals a day, 43.0% of women consumed two meals. Only 28.3% devoted exclusive time to eating, while many combined meals with serving customers (71.7%) and handling money (40.5%). With respect to cooking methods, 69.0% preferred fried foods, 38.8% baked foods, 37.8% grilled foods, and 34.9% steamed foods (Table 1).

### ISOLATION DUE TO COVID-19 AND SUPPORT RECEIVED

Overall, 95.6% of participants reported complying with mandatory quarantine measures, and 73.2% remained in isolation for more than 12 weeks. Furthermore, 77.0% reported receiving support during this period, which was more frequent among women (80.0%). Types of support included food assistance (55.1%), financial support (37.3%), and payment arrangements with landlords (15.0%) (Table 1).

Support mostly lasted ≤ 3 months (90.0%), with a higher proportion among women (92.4%). Additionally, 82 out of every 100 workers received support during mandatory isolation from a private university, and 11 out of every 100 received government support (Table 1).

### MODERATE/SEVERE FOOD INSECURITY

It was found that 44.1% of the sample presented MSFI in their households, and this condition was more prevalent among female workers (51.4%) (Table 1).

### DIETARY HABITS ASSOCIATED WITH FOOD INSECURITY

Statistically significant associations (p < 0.05) were identified MSFI and emotional state, length of time food remained at the vending site before consumption, and the number of meals a day. The prevalence of MSFI was 67.0% higher among individuals who reported that their emotional state affected food intake (PR = 1.67; 95%CI = 1.35-2.07); 1,28 times higher among those who kept food at their vending site ≤ 3 hours before consumption (PR = 2.28; 95%CI = 1.34-3.86); 2,44 times higher among those who consumed one meal a day (PR = 3.44; 95%CI = 1.96-6.04); and 1,69 times higher among those who consumed two meals a day (PR = 2.69; 95%CI = 1.57-4.49) (Table 2).

The prevalence of MSFI was significantly lower (p < 0.05) among individuals who had a defined eating schedule (PR = 0.81; 95%CI = 0.67-0.99) and among those who consumed their meals at the workplace (PR = 0.68; 95%CI = 0.57-0.81). Despite the lack of statistical significance, a higher prevalence of MSFI was found among participants who consumed three meals a day and who combined food consumption with customer service activities (Table 2).

### ISOLATION CONDITIONS AND SUPPORT VARIABLES ASSOCIATED WITH FOOD INSECURITY

A statistically significant association (p > 0.05) was identified between MSFI and making payment arrangements with landlords (PR = 2.29; 95%CI = 1.48-3.54). A lower prevalence of MSFI was observed among individuals who received financial support and government subsidies (Table 2).

### FACTORS CONTRIBUTING TO THE EXPLANATION OF MODERATE/SEVERE FOOD INSECURITY

The following factors contributed significantly (p < 0.05) to a higher prevalence of MSFI: lack of work permit (adjusted PR = 1.33; 95%CI = 1.01-1.74), mood state affecting food intake (adjusted PR = 1.59; 95%CI = 1.20-2.12), consuming one meal a day (adjusted PR = 2.84; 95%CI = 1.30-8.02), and making payment arrangements with landlords during the pandemic (adjusted PR = 2.19; 95%CI = 1.48-3.37) (Table 3).

**Table 3:** Conditions contributing to explain moderate/severe food insecurity among the working population during the pandemic (n = 656), Medellín 2021

Condition/characteristic	Crude PR	95%CI		Adjusted PR	95%CI	
		LT	UT		LT	UT
Age (two age groups) - 18-49 years	1.28	1.05	1.56	1.19	0.91	1.56
Sex - Female	1.34	1.13	1.60	1.27	0.96	1.59
Primary household income earner - Yes	1.05	0.99	1.12	1.00	0.81	1.17
Marital status - Without a partner	1.16	1.01	1.33	1.03	0.78	1.37
Work permit - No	1.26	1.08	1.47	1.33	1.01	1.74
Alcohol use - Yes	0.83	0.61	1.12	0.84	0.61	1.18
Tobacco use - Yes	1.37	1.00	1.88	1.23	0.88	1.76
Physical activity - Sedentary	1.06	0.85	1.39	1.17	0.88	1.56
Defined eating schedule - Yes	0.81	0.67	0.99	1.50	0.80	1.37
Consumes meals at the vending site - Yes	0.68	0.57	0.81	0.57	0.39	0.80
Mood state affects food intake - Yes	1.67	1.35	2.07	1.59	1.20	2.12
Food remains at the vending site before consumption - ≥ 3 h	2.28	1.34	3.86	2.14	0.97	6.10
Number of meals a day - One	1.74	1.39	2.19	2.84	1.30	8.02
When consuming meals						
Devotes exclusive time for eating - Yes	0.84	0.68	1.03	0.27	0.09	0.67
Combines meals with serving customers - Yes	1.15	0.94	1.41	0.39	0.15	1.03
Support during mandatory isolation						
Financial support - Yes	0.87	0.69	1.10	0.79	0.56	0.99
Payment arrangement with the landlord - Yes	2.29	1.48	3.54	2.19	1.48	3.37
Government support during the pandemic - Yes	0.89	0.78	1.01	0.82	0.59	1.22
Duration of government or private support - ≤ 1 month	1.18	0.97	1.46	1.24	0.90	1.75

LT = lower threshold; PR = prevalence ratio; UT = upper threshold.

Bold type denotes statistical significance.

In turn, the following factors contributed significantly (p < 0.05) to a lower prevalence of MSFI: consuming meals at the vending site (adjusted PR = 0.57; 95%CI = 0.39-0.80), devoting exclusive time to eating (adjusted PR = 0.27; 95%CI = 0.09-0.67), and receiving financial support during mandatory isolation (adjusted PR = 0.79; 95%CI = 0.56-0.99) (Table 3).

Despite the lack of statistical significance, the following conditions contributed to a higher prevalence of MSFI: being female, tobacco use, food remaining at the vending site ≥ 3 hours before consumption and receiving support during the pandemic for ≤ 1 month. After adjustment of the variables in the model, the following retained statistical significance (p < 0.05): lacking a work permit, consuming food at the workplace, mood affecting food intake, food remaining at the vending site for ≥ 3 hours before consumption, consuming one meal a day, and making payment arrangements with landlords during the pandemic. In turn, devoting exclusive time to eating and receiving financial support during mandatory isolation gained statistical significance (Table 3).

### FACTORS THAT CHARACTERIZE MODERATE/SEVERE FOOD INSECURITY PROFILE IN THE WORKING POPULATION

**Figure 2:**
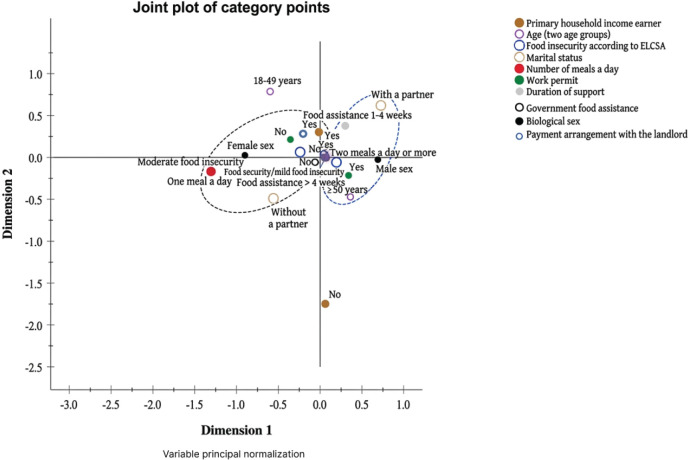
Profile of lifestyle factors and dietary and nutritional habits among informal workers from downtown Medellín during the pandemic, 2021. ELCSA = Latin American and Caribbean Food Security Scale.

The profile shown in Figure 2 reveals two subgroups, with a total explained variance of 2.82, Cronbach’s alpha of 0.366 for dimension 1 and 0.282 for dimension 2, and a mean of 0.326. The most relevant discrimination measures were: sex (0.619; dimension 1), primary household income earner (0.540; dimension 2), marital status (0.408; dimension 2), and age (0.292). The highest correlations were identified for not having a partner, being female, and being the primary household income earner.

The first and fourth quadrants of Figure 2 grouped characteristics that coincide with the position of the supplementary variable MSFI. These quadrants included: being female, consuming one meal a day, not having a partner, being the primary household income earner, lacking work permit, not receiving government food assistance during the pandemic, making payment arrangements with landlords, and receiving food assistance from a private university in the city (Figure 2).

The second and third quadrants included workers who had a partner, consumed ≥ two meals a day, had work permit, were male, received food assistance from 1 to 4 weeks during mandatory isolation, and were aged ≥ 50 years. These characteristics coincided with the position of the supplementary variable food security/mild food insecurity (Figure 2).

## DISCUSSION

The impact of the pandemic on food insecurity has been both global and local. Although no significant effects were observed on food supply, negative effects were identified regarding demand, particularly food access, mainly due to decline in income among most vulnerable populations [^18^]. The prevalence of MSFI in the working population in 2021 was 44.0%, a figure 7.3 percentage points higher among women (51.4%). However, in this same working population, the pre-pandemic prevalence of MSFI was 54.0%, reaching 61.5% among women [^4^].

According to data from the Food and Agriculture Organization of the United Nations, in 2021, approximately 30.0% of the world population experienced moderate and severe levels of food insecurity, corresponding to 2.37 billion people [^18^]. In the present study, the prevalence identified in this working population was 14 percentage points higher than that reported globally, highlighting their socio-occupational and environmental vulnerability, although it was 10 percentage points lower than the prevalence identified during the pre-pandemic period [^4^].

Contrary to these results, Kansiime et al. [^19^], when evaluating the implications of the pandemic on income and food security in Kenia and Uganda (regions with a high proportion of informal workers), observed that, compared with a typical period, the number of food insecure households increased by 38% and 44% in Kenia and Uganda, respectively. Likewise, compared with a typical period, severe food insecurity levels have been exacerbated by 7 and 20 percentage points among respondents in Uganda and Kenia, respectively.

Although the present study was conducted in the city of Medellín, it is important to highlight that food insecurity during the pandemic was documented across different populations in Latin America and the Caribbean. Indeed, PAHO reported that, in 2020, 267 million people experienced some form of food insecurity [^16^], with an undernourishment prevalence below 10.0% in Colombia, despite an ongoing upward trend [^16^].

### WORKERS’ SOCIODEMOGRAPHIC CONDITIONS

The largest proportion of workers was between 45 and 59 years, followed by those aged ≥ 60 years, indicating that within a few years this population will be predominantly composed of older adults. This situation, together with the low income levels reported in previous studies [^4^-^6^], is likely to contribute to poorer food security conditions, as noted by Estrada Restrepo et al. [^20^]. It is noteworthy that, although hombres predominated in this study, women were more frequently the primary household income earner, and most did not have a partner. These findings are consistent with data reported by the National Administrative Department of Statistics, which showed that women were the primary household income earner in 34.8% of households in 2016, increasing to 36.9% in 2018 [^21^]. Both internationally and in the present study, women exhibited a higher risk of severe food insecurity [^22^,^23^], contributing to previously described phenomena such as the feminization of poverty [^4^,^10^].

Workers’ biological sex, age, marital status, and lack of work permit were significant associated with the presence of MSFI. These sociodemographic characteristics have been consistently linked to food insecurity in different populations [^24^,^25^].

Empirical evidence indicates that female workers exhibit a higher prevalence of food insecurity compared with male workers in univariate analysis [^26^]. However, this association loses statistical significance in multivariate models [^27^], suggesting that the observed difference may be more closely related to contextual factors, such as lower income and being the primary household income earner, as evidenced in the present study. Nevertheless, among the female workers included in this study, these associations persisted and were strengthened in both dependent and interdependent multivariate analyses.

Lack of work permit was significantly associated with MSFI, a finding that is difficult to compare with previous evidence due to the limited literature examining this condition among subsistence workers operating on urban streets and sidewalks.

### WORKERS’ LIFESTYLES AND DIETARY HABITS

Most workers reported not having either an exclusive time or space for food consumption and combined eating with customer service and handling of money; practices that have been documented in previous studies [^4^-^6^] and that, in the present study, were associated with a higher prevalence of food insecurity.

A preference for high-fat foods and increased frequency of food consumption was observed, findings similar to those reported in previous studies conducted in the same population [^4^-^6^]. These factors may contribute to explaining the higher prevalence of overweight and obesity [^7^] in this population compared with that observed in Medellín [^28^].

Mood state, reflected in levels of stress, anxiety, and depression, affected food consumption and was associated in the multivariate model with the prevalence of MSFI, as had already been observed before the pandemic [^4^], This suggests that mood-related food choices may represent an indicator of deteriorating mental health conditions among workers, as food insecurity was exacerbated by COVID-19 lockdowns and restrictions. Similar findings have also been reported in a study conducted in rural areas of Bangladesh [^29^]. However, the cross-sectional design of the present study precludes establishing a causal relationship between mental health and MSFI.

### SUBSIDIES, FINANCIAL SUPPORT, AND DURATION OF SUPPORT

In order to mitigate the effects of the novel coronavirus pandemic, governments implemented several social assistance programs to enable the most vulnerable populations to cope with the new challenges imposed by the public health emergency.

In the present study, 77.0% of informal workers received support, primarily in the form of food assistance, often for periods shorter than 3 months. Furthermore, a lower prevalence of MSFI was observed among those who received financial support and among those who reported receiving subsidies from a private university and from the government. However, these differences were not statistically significant in either the bivariate or multivariate analysis. These findings are consistent with those of 2020 study on food insecurity in Canadian households [^25^], in which, although the prevalence of food insecurity was significantly higher among households receiving charitable food assistance, no statistically significant differences were observed in the severity of food insecurity between food-insecurity households receiving charitable food assistance and those not receiving such assistance.

Although dietary habits and lifestyles were explored in this working population during the pandemic period (2020-2021), food consumption frequencies according to food type were not reported. In previous studies [^7^,^16^-^18^], this information has enabled additional characterization of the vulnerability profile observed during the early stages of COVID-19 pandemic and should therefore be included in future analyses of the nutritional and dietary components of this working population.

In this same population during the pre-pandemic period [^4^], the conditions that explained MSFI were alcohol consumption and having an exclusive time for food consumption. In turn, the conditions that characterized this food insecurity profile were: not consuming alcohol, mood affecting food intake, consuming one or two meals a day, lacking a defined eating schedule, not having work permit, and being female [^4^]. These conditions were similar to those identified in the present study, in which receiving subsidies during mandatory isolation for more than 4 weeks and not having a partner also contributed to characterizing the profile of MSFI.

A limitation in the analysis of some variables related to mandatory isolation and support received during the pandemic is the scarcity of literature focused on this population group. However, this gap may gradually be addressed through studies such as the present one, which seeks to contribute to the understanding and characterization of food security among subsistence workers and their households during pandemic periods. Such evidence may help support the planning of actions aimed at this working population in public health emergencies and other exceptional circumstances requiring timely and appropriate responses, while considering other studies and investigations conducted in Medellín, Colombia.

## CONCLUSIONS

This study provided evidence of the high prevalence of MSFI among informal workers in downtown Medellín during the pandemic. Nevertheless, a 10-percentage-point decrease was observed compared with the pre-pandemic period, although conditions among women worsened. Factors that characterized the profile of MSFI included being female, consuming one meal a day, not having a partner, being the primary household income earner, having work permit, not receiving food assistance from the government during the pandemic (including mandatory isolation, making payment arrangements with landlords during the pandemic, and receiving food assistance from a private university.

## Data Availability

The data supporting the findings of this study are available within the article
